# Left cardiac sympathetic denervation for the management of life-threatening ventricular tachyarrhythmias in young patients with catecholaminergic polymorphic ventricular tachycardia and long QT syndrome

**DOI:** 10.1007/s00392-012-0492-7

**Published:** 2012-07-21

**Authors:** Heike E. Schneider, Michael Steinmetz, Ulrich Krause, Thomas Kriebel, Wolfgang Ruschewski, Thomas Paul

**Affiliations:** 1 Department for Pediatric Cardiology and Intensive Care Medicine, Georg-August-University Göttingen, Robert-Koch-Str. 40, 37075 Göttingen, Germany; 2 Department for Thoracic and Cardiovascular Surgery, Georg-August-University Göttingen, Robert-Koch-Str. 40, 37075 Göttingen, Germany

**Keywords:** CPVT, Long QT syndrome, Left cardiac sympathetic denervation, Children and young adults

## Abstract

**Background:**

Left cardiac sympathetic denervation (LCSD) may be a therapeutic adjunct for young patients with catecholaminergic polymorphic ventricular tachycardia (CPVT) and long QT syndrome (LQTS) who are not fully protected by beta-blockade.

**Objective:**

The objective of this analysis was to report our institutional experience with LSCD in young patients for the management of life-threatening ventricular arrhythmias in CPVT and LQTS.

**Methods:**

Ten young patients with CPVT and LQTS underwent transaxillary LSCD at our institution. Mean age at surgery was 14.0 (range 3.9–42) years, mean body weight was 45.7 (range 15.5–90) kg. Five patients had the clinical diagnosis of CPVT, three were genotype positive for a mutation in the ryanodine-receptor-2-gene. Four of five LQTS patients were genotype positive. Indications for LCSD were recurrent syncope, symptomatic episodes of ventricular tachycardias and/or internal cardioverter–defibrillator (ICD) discharges, and aborted cardiac arrest despite high doses of beta-blockers.

**Results:**

LCSD was performed via the transaxillary approach. No significant complications were observed. Two patients already had an ICD, 6 patients received an ICD at the same operation or shortly thereafter. Median length of follow-up after LCSD was 2.3 (range 0.6–3.9) years. After LCSD a marked reduction in arrhythmia burden and cardiac events was observed in all patients while medication was continued. None of the patients had any further ICD discharge for sustained VT.

**Conclusions:**

After LCSD, arrhythmia burden could significantly be reduced in all our young patients with CPVT and LQTS.

## Introduction

Catecholaminergic polymorphic ventricular tachycardia (CPVT) and long QT syndrome (LQTS) are ion channelopathies of genetic origin characterized by life-threatening ventricular arrhythmias, which are triggered by increased sympathetic activity, for example, physical exercise and emotional stress. They often manifest at young age, the first event may be fatal [1–6].

Therefore, in order to prevent cardiac events, the cornerstone of medical treatment for patients with CPVT and LQTS is beta-blocker pharmacotherapy [4–6]. However, certain patients remain symptomatic despite high doses of beta-blockers [7–10]. Particularly, 46–69 % of patients with CPVT have been reported to continue to have cardiac events [2]. In addition, some patients do not tolerate high-dose beta-blockade. Finally, in teenage years poor compliance with daily medication is known in patients with chronic diseases and these patients often become symptomatic again during this period of life. ICD implantation is recommended in drug refractory, highly symptomatic cases [11]. However, fatal failure of ICD therapy has been reported [12] and frequent appropriate or inappropriate ICD discharges as well as electrical storms are responsible for significant morbidity and mortality and severely decreased quality of life [13].

Left cardiac sympathetic denervation (LCSD) has been suggested in this setting. Reports have documented its efficacy for LQTS patients [14–17] and also in three small case series for CPVT [16–18]. When performed appropriately, LCSD is an antifibrillatory intervention that largely prevents norepinephrine release in the heart without reducing heart rate [19–24]. In the present report, we describe our experience with LCSD for highly symptomatic young patients with LQTS and CPVT that were not fully protected by beta-blocker therapy.

## Patients and methods

### Patients

All 10 patients who had been treated with LCSD at the Children’s Heart Center Göttingen between October 2007 and November 2011 were included. Medical chart review was performed to obtain patient data, operative notes and perioperative course as well as mid-term follow-up. Chart review revealed five patients with the diagnosis of CPVT and five patients with LQTS. Four patients diagnosed with LQTS were tested positive for a mutation in the LQT 1-, LQT 3-, and LQT 8-gene, respectively. In three patients with CPVT a mutation in the *RYR*2-encoded cardiac ryanodine receptor/calcium release channel had been found. In the remaining two patients the results of genetic testing were negative. All patients had a structurally normal heart and normal left ventricular function.

Indications for LCSD were severe symptoms not adequately controlled by medical therapy including high-dose beta-blockade in all patients. Cardiac events included recurrent syncope, frequent non-sustained VT, sustained VT with or without stress, repeated appropriate ICD shocks/electric storm with multiple shocks, and aborted cardiac arrest. An ICD had already been implanted before LCSD in 2 patients, 6 patients received an ICD at the same operation or shortly thereafter (Table 1).

**Table 1:** Demographics, patient data and indication for left cardiac sympathetic denervation

Patient	Gender	Age at LCSD (years)	Weight at LCSD	Diagnosis	Failed medications	Reason for LCSD	ICD (yes/no)	Follow-up (years)
1	M	12.4	47.9	CPVT	Propranolol, metoprolol	Sustained VT, frequent ICD discharges and ICD storm	Yes, before LCSD	3.9
2	F	42	73.0	CPVT	Nadolol, verapamil	Multiple ICD discharges for VT, side effects of medication	Yes, before LCSD	2.6
3	M	17.0	90.0	CPVT	Metoprolol	Recurrent VT	Yes	2.3
4	M	9.3	41.8	CPVT	Metoprolol	Recurrent syncope	Yes	0.9
5	F	12.2	40.8	CPVT	Metoprolol	Recurrent syncope, aborted cardiac arrest	Yes, at time of LCSD	0.45
6	M	15.9	46.0	LQT 8	Propranolol	Recurrent VT, syncope, side effects of medication	Yes, at time of LCSD	1.2
7	F	16.3	58.0	LQT 3	Metoprolol	Recurrent syncope and seizures, side effects of medication	Yes, at time of LCSD	4.3
8	M	4.1	16.1	LQT 1	Propranolol	Recurrent syncope, seizures	No	1.2
9	M	3.9	15.5	LQT 1	Propranolol	Recurrent syncope	No	0.7
10	M	5.9	18.6	LQTS	Propranolol, esmolol, lidocaine	Aborted cardiac arrest	Yes, at time of LCSD	0.6
Median		15	57					2.3
Mean		14.02	45.7					1.8
Range		3.9–42	15.5–90					0.6–3.9

*CPVT* catecholaminergic polymorphic VT, *ICD* internal cardioverter/defibrillator, *LCSD* left cardiac sympathetic denervation, *LQTS* long QT syndrome, *VT* ventricular tachycardia

Values of sympathetic activity were obtained from ECG and Holter monitoring studies during the hospital stay unless stated otherwise.

The study protocol had been approved by the institutional scientific committee of the Heart Center Göttingen.

### Technique of surgical LCSD

The procedure was performed under general anesthesia in all patients. For the transaxillary approach a small approximately 3–4 cm wide incision was made just below the axillary hair line. During preparation care was taken to avoid injury to the lymphatic vessels. The pleural space was opened and the lung was retracted anteriorly in a fashion to allow exposure of the stellate and the second ganglion. The stellate ganglion was freed from its bed and the sympathetic chain could be followed down as far as the 4th–5th thoracic ganglion. The stellate ganglion was dissected in two halves, thus separating the first thoracic part from the inferior cervical part. The cephalic portion of the stellate ganglion was thus preserved to prevent Horner syndrome. Then the thoracic ganglia T2–T4/T5 and their associated rami communicantes were removed. The dissected material was sent to the pathology laboratory to ensure that nerve and ganglia were removed. A chest tube was inserted until the next morning.

### Statistical analysis

Continuous data are expressed as mean value ± standard deviation or median and range.

## Results

### Main findings

Mean age of the patients was 14 (range 3.9–42) years, mean body weight 45.7 (range 15.5–90) kg. Seven patients were male. Two patients already had an ICD implanted prior to LCSD. Four patients received an ICD at the same operation, an ICD was implanted shortly after LCSD in 2 patients (Table 1). In the two remaining patients, both with LQTS type 1, parents opted against the recommendation of ICD implantation after discussion of potential benefits and complications.

**Fig. 1:**
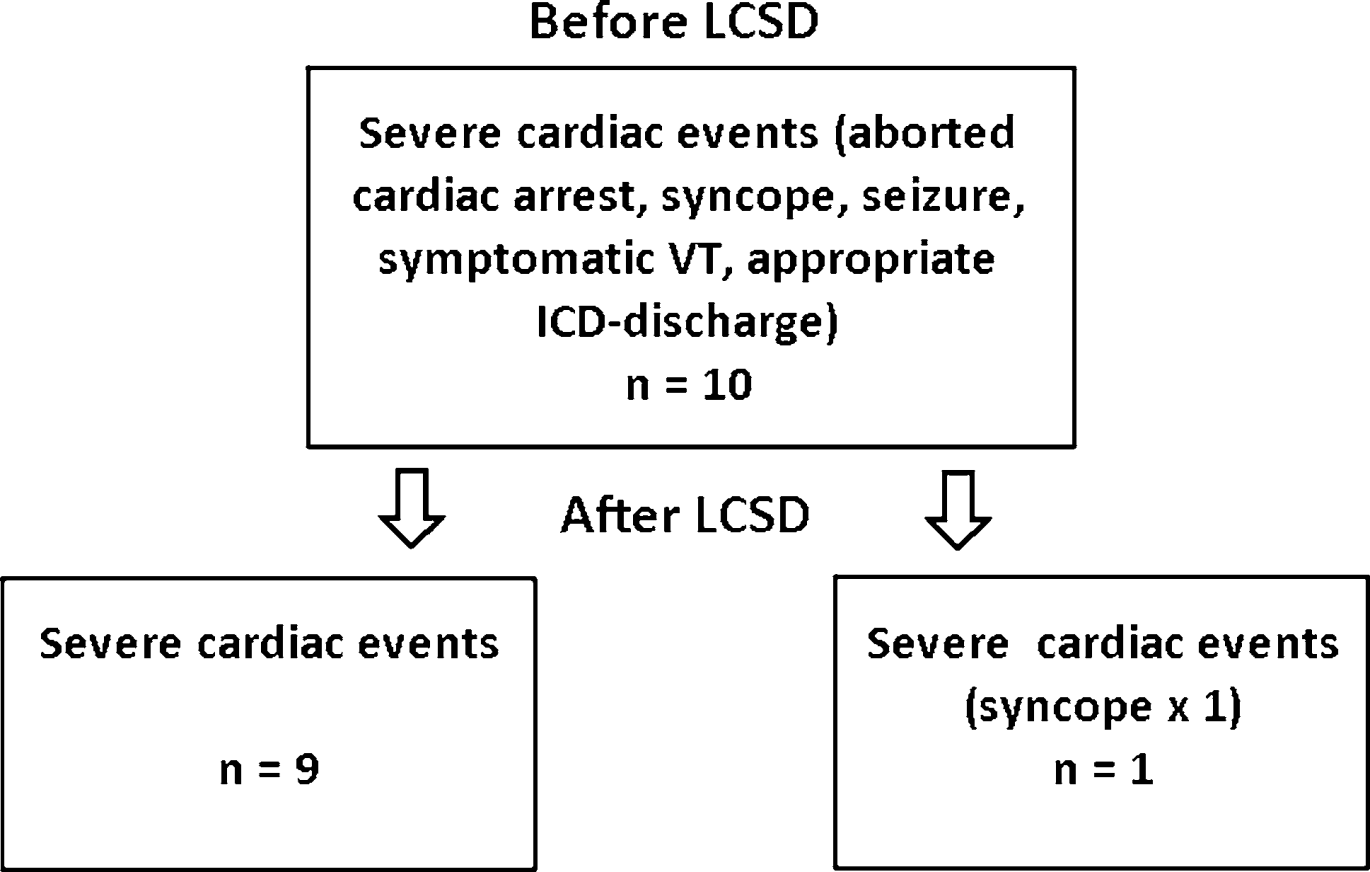
Graph illustrating reduction in severe cardiac events within our cohort. Severe cardiac events ranged from numerous VT episodes and ICD discharges in the two patients who already had an ICD, aborted cardiac arrest in 2 further patients, two recurrent seizures and syncope despite beta-blocker therapy in the youngest patient before left cardiac sympathetic denervation

The median length of follow-up after the procedure was 2.3 years (range 0.6–3.9) years. All patients had significant and persistent relief from their cardiac symptoms (Fig. 1).

### Perioperative course

Hospital stay for patients who received LCSD varied depending on the condition the patient was in when admitted to our unit. The two patients after cardiac arrest had a prolonged hospital stay of 2.5 and 8 weeks unrelated to LCSD. In the remaining 8 patients hospital stay ranged from 3 to 9 days. The patient that was hospitalized for 9 days had a pneumothorax after transvenous ICD implantation. Two other patients, one who also had ICD implant at the same time as LCSD, had a small pleural effusion that resolved spontaneously. In 7 of 10 patients we observed a mild Horner syndrome directly after LCSD which had resolved in all but one patient at the first follow-up visit. No major complications were noted in any of our patients.

### Outcome of patients with CPVT

All five patients with CPVT were continued on their medication with beta-blockade. None of them had any further appropriate ICD discharge since LCSD.

**Fig. 2:**
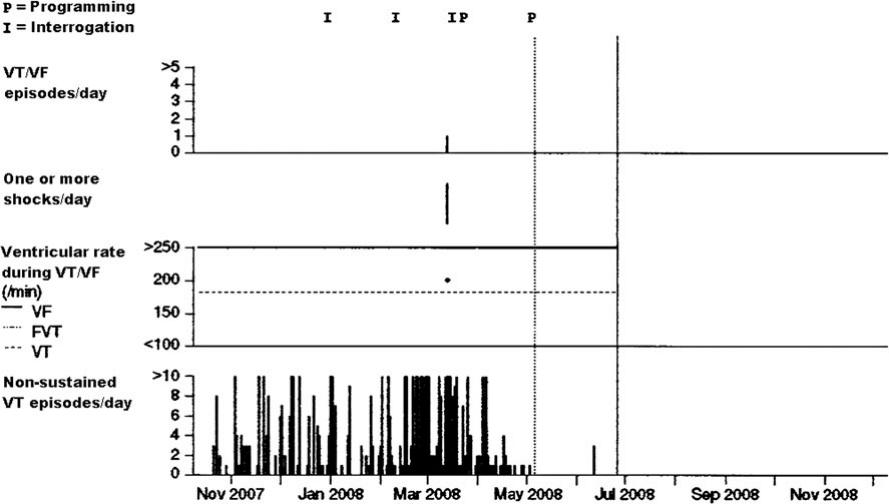
ICD recording of a 12-year-old boy with catecholaminergic ventricular tachycardia with frequent ICD discharges: following left cardiac sympathetic denervation in May 2008 (*dotted line*) a marked reduction of episodes of ventricular tachycardia is evident. No further ICD discharge has occurred up to date

Patient 1 (Table 1) had the most dramatic improvement after the procedure. Despite high-dose beta-blockade (6 mg/kg propranolol), this boy continued to have frequent episodes of sustained VT resulting in appropriate ICD discharges. From the day of LCSD, while he was maintained on the same high dose of propranolol, these episodes subsided (Fig. 2). After more than 2 years, however, when he became incompliant with his medication, ICD telemetry again exhibited frequent non-sustained VT episodes, which resolved after resuming his prescribed medical regimen.

**Fig. 3:**
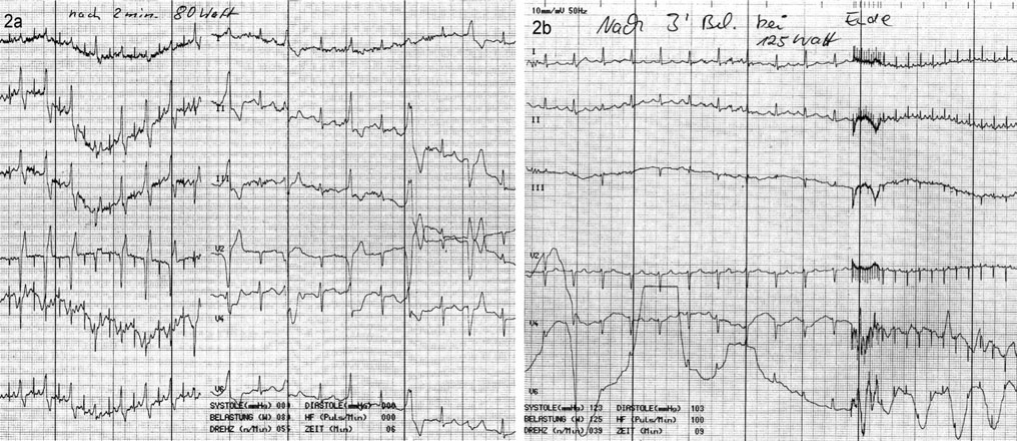
Six-lead electrocardiograms (ECGs) obtained from patient #2 during exercise stress testing before and 2 days after left cardiac sympathetic denervation. **a** ECG at a work load of 80 W for 2 min: frequent ventricular premature beats are present as ventricular bigemini with 2 different morphologies. The test was terminated. **b** Sinus rhythm is still present at 125 W for 3 and a half minutes without any ventricular ectopy. The patient terminated the test at this level due to muscular exhaustion

Patient 2 also had frequent ICD discharges while on therapy with beta-blockade (60 mg nadolol) that slightly decreased in number after addition of calcium channel blockers [7–9], but did not subside. Exercise stress testing performed before and shortly after LCSD showed significantly decreased arrhythmia burden (Fig. 3). Since LSCD, no further appropriate ICD discharge has occurred while medications could slightly be tapered.

The third patient with CPVT was a 17-year-old boy, who chose LCSD after recurrent episodes of syncope on beta-blockers. Prior to LCSD and despite high-dose metoprolol (150 mg) fast polymorphic VT could reproducibly be induced during exercise stress tests. Repeat exercise stress testing 3 days after LCSD showed marked reduction in ventricular ectopy. Non-sustained VT episodes are regularly recorded by the ICD and a near syncopal event occurred. As he does not show for regular follow-up visits, this event was attributed to non-compliance with his medical regimen.

The fourth patient with CPVT, a 9-year-old boy, presented with recurrent syncope during exercise despite high-dose beta-blocker therapy. After LCSD he did not experience any further syncope.

Finally, the fifth patient of this group was referred to our institution after an aborted cardiac arrest while on metoprolol. Prior to this event, recurrent syncope with exercise had occurred and exercise stress testing had documented an increase in ventricular ectopy. For secondary prevention, an ICD was implanted and LCSD performed. ICD telemetry has not shown any sustained or non-sustained episodes of VT since LCSD.

### Outcome of patients with LQTS

Following LCSD, no significant shortening of the QTc interval could be observed in any of our LQTS patients (Table 2).

**Table 2:** Sympathetic activity before and after left cardiac sympathetic denervation

Patient	Diagnosis	Change mean heart rate Holter (/min)	Change PR interval (ms)	QTc interval (ms)		Change systolic blood pressure (mmHg)	Heart rate variability SDNN pre (ms)	Heart rate variability SDNN post (ms)	Heart rate variability rMSDD pre (ms)	Heart rate variability rMSDD post (ms)
				Pre	Post					
1	CPVT	–	−10	401	395	+2	Normal^a^	–	Normal^a^	–
2	CPVT	−1	−10	417	412	−2	82	48	62	35
3	CPVT	+31	−15	436	444	+4	255	–	188	–
4	CPVT	−7	+10	370	388	+1	258	240	175	171
5	CPVT	+6	+10	359	406	0	133	77	72	60
6	LQT 8	+6	0	535	510	+3	207	173	141	70
7	LQT 3	–	20	409	410	+1	174	Normal^a,b^	92	Normal^a,b^
8	LQT 1	−4	−10	516	516	+2	108	82	36	43
9	LQT 1	+10	0	465	472	+7	132	95	59	38
10	LQTS	–	−7	474	459	+4	–	40	–	13

*ms* milliseconds, – no data available

^a^Other measures of heart rate variability

^b^Obtained at follow-up visit

This boy (patient #6, Table 1) had been followed for cerebral seizures until the age of 6 years when LQTS was diagnosed. Beta-blocker therapy was then initiated and a mutation was found in the LQT 8-gene. Despite increasing doses up to 5 mg/kg propranolol he continued to experience syncopal episodes followed by seizures. In addition, he exhibited fatigue likely due to decreased heart rate und hypotension as side effects of high-dose propranolol. At the time of LCSD, an ICD was also implanted. Since then, no further episodes of syncope have occurred while his dose of propranolol could be tapered to 3 mg/kg per day. ICD analysis during follow-up did not show any significant episodes of ventricular ectopy.

A 16-year-old girl with LQT 3 still developed non-sustained polymorphic VT during exercise stress testing while on daily metoprolol (3 mg/kg). Since LCSD and more than 3 years of follow-up, no syncope or ICD discharge occurred. ICD telemetry revealed only 2 short non-sustained runs of VT in the last 6-month period.

Two patients (#8 and #9) had a mutation in the LQT 1 gene. These were the youngest patients within our group, 3 and 4 years of age, respectively. Both had recurrent syncopal episodes, one also with seizures, despite high doses of propranolol (4 and 5 mg/kg per day, respectively). Since LCSD, no syncope has occurred while both were continued on the same medication as before. In each case, implantation of an ICD was declined by the parents.

The last patient (#10) with LQTS, who was mutation negative for LQT types 1–3, received LCSD at time of ICD implantation for his second aborted cardiac arrest while on beta-blocker therapy.

### Sympathetic activity

None of our patients had a decrease in systolic blood pressure or mean heart rate on Holter recording after LCSD. The corrected QT interval (according to Bazett’s formula) was normal in our CPVT patients before LCSD, after the procedure it decreased in two and increased in duration insignificantly in three patients. The corrected QT interval in patients with LQTS decreased slightly but remained abnormal in two patients, remained the same in two patients and increased in one patient. The PR interval exhibited a certain degree of variability with an increase up to 20 ms and a decrease up to 15 ms within our patient cohort. Using the standard deviation of all normal R–R intervals of the Holter recording (SDNN) as a measure of heart rate variability, we observed a decrease in all eight patients in whom complete data were available. Two patients had a low heart rate variability (<50 ms) after the procedure, one had had a recent aborted cardiac arrest (Patient #10) and the other had already shown a decreased heart rate variability (<100 ms) before the procedure. Heart rate variability with focus on the parasympathetic branch showed also a decrease in the majority of our patients but was clearly abnormal only in the patient after a recent aborted cardiac arrest (Patient #10), for details please see Table 2.

## Discussion

### Main findings

We report our experience of LCSD in nine children and one young adult with diseases associated with significant risk of sudden cardiac death, CPVT and LQTS. LCSD decreased arrhythmia burden significantly in all patients, but not all patients exhibited the same amount of benefit (Fig. 1). Patient (#1, Table 1) with CPVT demonstrated the most symptomatic improvement after LCSD but showed recurrence of VT episodes during incompliance with his beta-blocker therapy in adolescence. VT disappeared again after resuming his high-dose regimen, and the positive effects persist. ICD telemetry in the 17-year-old boy (#3, Table 1) after a near syncopal event occasionally documented non-sustained episodes of VT. Similarly to patient #1, we assume that this adolescent was not compliant with his medication because he also had not regularly shown for scheduled follow-up visits. This implies that LCSD alone may not be sufficient to completely suppress VT in certain patients with CPVT. Therefore, in patients with CPVT the same dose of beta-blockade should be maintained to avoid recurrent symptoms.

On the other hand, in one of our patients with LQTS and side effects of beta-blocker therapy, medication could be reduced after LCSD. No cardiac events and no significant VT episodes on ICD interrogation have been noted in the follow-up period. This underscores that dosage of beta-blockers has to be chosen on an individual basis for the personalized medicine requirements of patients with LQTS and CPVT [17, 25–28].

Several small studies have suggested that a more cardioselective beta-blocker, such as atenolol, may be less efficacious than non-selective beta-blockers such as propranolol and nadolol in treatment of symptomatic patients with LQTS [29]. Patients with LQT1 benefit the most from beta-blocker therapy when compared to those with LQT2 and LQT3. One study showed that only 10 % LQT1 of patients had breakthrough symptoms while 23 % of LQT2 and 32 % of LQT3 patients had breakthrough [30]. Controversy exists for treatment of LQT3 with beta-blockers as they may be potentially proarrhythmic because of concomitant slowing of heart rate. As LCSD has been shown not to decrease heart rate [19–24], as also observed in our small cohort, LCSD may be a particular good option in patients with LQT3. Among genotyped LQTS patients, LCSD tended to be significantly more effective in patients with LQT1 and LQT3 [15].

### Surgical technique

A minimally invasive approach, video-assisted thoracoscopic cardiac denervation, has recently been described in two case series [16, 17]. This technique offers a smaller operative trauma than the open surgical transaxillary approach [31] as used in our institution. An open surgical approach via supraclavicular exposure [32] has been applied in the largest series published by Schwartz et al. [15] without major complications.

The transaxillary approach [31] leads to a good exposure of the sympathetic trunk, intercostal and subclavian arteries, the thoracic duct and phrenic nerve. In particular, it allows good control of accidental bleeding from the intercostal vessels while life-threatening great vessel injury is rare. Especially in infants, small children or patients with a short neck, the transaxillary approach may be a good surgical option versus the thoracoscopic approach [16, 17, 31–33].

No significant complications were noted in the immediate operative and postoperative period in our patients which may include an electrical storm [16]. In most of our patients incomplete Horner syndrome was observed transiently, likely due to compression and swelling of the upper half of the stellate ganglion. In 9/10 patients unilateral Horner syndrome had disappeared at last follow-up. Neurologic symptoms, such as neuralgia and sharp arm pain [32], or harlequin facial flushing up to several years [16], were not observed in our patient group.

Nevertheless, no matter which technique of LCSD was used, perioperative complications were minor and significant symptomatic relief for the affected patients after LCSD has uniformly been observed.

Recently, beneficial effects of catheter-based renal sympathetic denervation for the treatment of patients with heart failure and malignant tachyarrhythmias have been reported. This new, still experimental approach might offer the opportunity to achieve reduction of the cardiac sympathetic activity even without an operation [34].

### Management approach in CPVT patients

As experience of LCSD in CPVT patients is limited, and a new treatment option with flecainide has been reported recently, these patients deserve additional comments.

In our small cohort of patients with CPVT, LCSD was very effective in preventing new life-threatening tachycardia events. In the past, calcium channel blocking agents have been used in patients with ineffective β-blocker therapy [7–9]. However, in subsequent reports calcium channel blocking agents were not consistently effective in preventing sudden cardiac death in CPVT patients [7, 35, 36]. It is of note that also two of our patients had been treated unsuccessfully with verapamil.

Flecainide has been reported recently in a multicenter case series to be effective in 75 % of the patients to reduce exercise-induced ventricular arrhythmias [37]. A plausible molecular mechanism and an animal model support its potential as a useful drug in CPVT [38, 39]. It has been suggested that flecainide in addition to beta-blocker therapy should be considered for CPVT patients who otherwise have few alternative therapeutic options. Although data seem very promising, as number of patients and mean follow-up were limited to 20 months, these findings need to be validated in a larger group of patients with a prolonged follow-up.

It may be speculated that some patients with CVPT and persistent symptoms under beta-blocker therapy will benefit from flecainide without the need of LCSD. It is of note, however, that medical therapy will only be effective as long as the patients adhere to strict regular drug intake with an effective dose. An appropriate ICD discharge was observed in one patient who had a low flecainide level in the study reported by van der Werf et al. [37]. As with any oral medication, the problems of regular intake and poor compliance are still of paramount importance. In addition, a sufficient dose of flecainide has to be achieved with the potential of side effects as this drug has a relatively narrow therapeutic range. Finally, one has to keep in mind, that in case of treatment failure, the first event may be fatal. Depending on the individual situation of the patient, flecainide in addition to beta-blocker therapy alone may not be safe in every patient to prevent syncope and sudden cardiac death. Therefore, we think, that LCSD should be offered to young patients with CPVT who are not sufficiently protected by medical therapy.

### Outlook

LCSD is shown to be beneficial for highly symptomatic patients with CPVT and LQTS. Despite these favorable results [40], LCSD has not yet found its place in routine or escalating care of patients with drug refractory LQTS and CPVT [41, 42]. Schwartz [25] has recently proposed that LCSD may even alter the necessity of ICD implantation in selected patients, in particular LQT 1 patients [26], who currently meet the class I indication for ICD implantation as adverse events after ICD implantation have been reported to be as high as 25 % in young patients [13].

In addition, in patients with CPVT not sufficiently protected by medical therapy with an indication for ICD implantation, combined LCSD at time of ICD implant [25] has the potential to prevent ICD discharges. In this special group of patients the first ICD shock—appropriate or not—may trigger a sympathetic surge with subsequent shocks or even an electrical storm.

### Limitations

Limitations of this study are due to the inherent nature of a retrospective design involving a limited patient number secondary to the rare prevalence of these diseases. Quantitative marker of arrhythmia burden has been difficult to express in statistically comparable data due to the large variability in presenting symptoms. While benefits of LCSD for LQTS patients have been described in long-term follow-up [15], particular interest belongs to the short- and long-term outcome of patients with CPVT, as to our knowledge, our reports adds 5 more patients, up to a total number of 27 patients published yet, in whom LCSD uniformly has been performed successfully to diminish significant arrhythmogenic cardiac events and appropriate ICD discharges ([16–18, 25, 43–46]; Table 3). Data from a larger number of patients are needed to further assess the effects of LCSD for CPVT and LQTS patients.

**Table 3:** Number and characteristics of previously published patients with catecholaminergic polymorphic ventricular tachycardia that underwent left cardiac sympathetic denervation

Reference	Number of patients reported (*N*)	Gender (M)	Follow-up	Significant improvement of symptoms (*N*)
Wilde et al. [18]	3	2	30 months–20 years	3
Atallah et al. [16]	4	2	1 month–2 years	3, 1 had no follow-up
Scott et al. [45]	1	0	4 years	1, bilateral CSD
Collura et al. [17]	2	0	15 months	2
Makanjee et al. [46]	1	0	10 years	1
Gopinathannair et al. [44]	1	0	16 months	1
Odero et al. [32]	14, personal communication	No details provided	No details provided	13
Moray et al. [43]	1	1	–	–
Total number	27		0.1–20 years	24

*M* male, *N* number, *CPVT* catecholaminergic polymorphic VT, *ICD* internal cardioverter/defibrillator, *LCSD* left cardiac sympathetic denervation, *LQTS* long QT syndrome, *VT* ventricular tachycardia

## Conclusion

LCSD via the transaxillary approach could safely be performed with only minimal acute and long-term complications. LCSD effectively decreased the arrhythmia burden in children and young adults with CPVT and LQTS. In particular, LCSD decreased appropriate ICD discharges in patients with CPVT.

## Abbreviations

CPVT: Catecholaminergic polymorphic ventricular tachycardia
ICD: Internal cardioverter-defibrillator
LCSD: Left cardiac sympathetic denervation
LQTS: Long QT syndrome
VT: Ventricular tachycardia

## References

[1] Leenhardt A, Lucet V, Denjoy I, Grau F, Ngoc DD, Coumel P (1995). Catecholaminergic polymorphic ventricular tachycardia in children: a 7-year follow-up of 21 patients. Circulation.

[2] Priori SG, Napolitano C, Memmi M, Colombi B, Drago F, Gasparini M, DeSimone L, Coltorti F, Bloise R, Keegan R, Cruz Filho FE, Vignati G, Benatar A, DeLogu A (2002). Clinical and molecular characterization of patients with catecholaminergic polymorphic ventricular tachycardia. Circulation.

[3] Ackerman MJ (2004). Cardiac channelopathies: it’s in the genes. Nat Med.

[4] Napolitano C, Priori SG (2007). Diagnosis and treatment of catecholaminergic polymorphic ventricular tachycardia. Heart Rhythm.

[5] Goldenberg I, Moass AJ (2008). Long QT syndrome. J Am Coll Cadiol.

[6] Moss AJ, Zareba W, Hall WJ, Schwartz PJ, Crampton RS, Benhorin J, Vincent GM, Locati EH, Priori SG, Napolitano C, Medina A, Zhang L, Robinson JL, Timothy K, Towbin JA, Andrews ML (2000). Effectiveness and limitations of beta-blocker therapy in congenital long-QT syndrome. Circulation.

[7] Sumitomo N, Harada K, Nagashima M, Yasuda T, Nakamura Y, Aragaki Y, Saito A, Kurosaki K, Jouo K, Koujiro M, Konishi S, Matsuoka S, Oono T, Hajakawa S, Miura M, Ushinohama H, Shibata T, Niimura I (2003). Catecholaminergic polymorphic ventricular tachycardia: electrocardiographic characteristics and optimal therapeutic strategies to prevent sudden death. Heart.

[8] Swan H, Laitinen P, Kontula K, Toivonen L (2005). Calcium channel antagonism reduces exercised-induced ventricular arrhythmias in catecholaminergic polymorphic ventricular tachycardia patients with RYR2 mutations. J Cardiovasc Electrophys.

[9] Rosso R, Kalman JM, Rogowski O, Diamant S, Birger A, Biner S, Belhassen B, Viskin S (2007). Calcium channel blockers and beta-blockers versus beta-blockers alone for preventing exercise-induced arrhythmias in catecholaminergic polymorphic ventricular tachycardia. Heart Rhythm.

[10] Krause U, Gravenhorst V, Kriebel T, Ruschewski W, Paul T (2011). A rare association of long QT syndrome and syndactyly: Timothy syndrome (LQT 8). Clin Res Cardiol.

[11] Zipes DP, Camm AJ, Borggrefe M, Buxton AE, Chaitman B, Fromer M et al., American College of Cardiology/American Heart Association Task Force, European Society of Cardiology Committee for Practice Guidelines, European Heart Rhythm Association, Heart Rhythm Society (2006) ACC/AHA/ESC 2006 Guidelines for Management of Patients With Ventricular Arrhythmias and the Prevention of Sudden Cardiac Death: a report of the American College of Cardiology/American Heart Association Task Force and the European Society of Cardiology Committee for Practice Guidelines (writing committee to develop Guidelines for Management of Patients With Ventricular Arrhythmias and the Prevention of Sudden Cardiac Death): developed in collaboration with the European Heart Rhythm Association and the Heart Rhythm Society. Circulation 114:e385–484 10.1161/CIRCULATIONAHA.106.178233 16935995

[12] Mohamed U, Gollob MH, Gow RM, Krahn AD (2006). Sudden cardiac death despite an implantable cardioverter–defibrillator in a young female with catecholaminergic ventricular tachycardia. Heart Rhythm.

[13] Schwartz PJ, Spazzolini C, Priori SG, Crotti L, Vicentini A, Landolina M (2010). Who are the long-QT syndrome patients who receive an implantable cardioverter–defibrillator and what happens to them? Data from the European Long-QT Syndrome Implantable Cardioverter-Defibrillator (LQTS ICD) Registry. Circulation.

[14] Schwartz PJ, Locati EH, Moss AJ, Crampton RS, Trazzi R, Ruberti U (1991). Left cardiac sympathetic denervation in the therapy of congenital long QT syndrome. A worldwide report. Circulation.

[15] Schwartz PJ, Priori SG, Cerrone M, Spazzolini C, Odero A, Napolitano C, Bloise R, De Ferrari GM, Klersy C, Moss AJ, Zareba W, Robinson JL, Hall WJ, Brink PA, Toivonen L, Epstein AE, Li C, Hu D (2004). Left cardiac sympathetic denervation in the management of high-risk patients affected by the long-QT syndrome. Circulation.

[16] Atallah J, Fynn-Thompson F, Cecchin F, DiBardino DJ, Walsh EP, Berul CI (2008). Video-assisted thoracoscopic cardiac denervation: a potential novel therapeutic option for children with intractable ventricular arrhythmias. Ann Thorac Surg.

[17] Collura CA, Johnson JN, Moir C, Ackerman MJ (2009). Left cardiac sympathetic denervation for the treatment of long QT syndrome and catecholaminergic polymorphic ventricular tachycardia using video-assisted thoracic surgery. Heart Rhythm.

[18] Wilde AAM, Bhuiyan ZA, Crotti L, Facchini M, De Ferrari GM, Paul T, Ferrandi C, Koolbergen DR, Odero A, Schwartz PJ (2008). Left cardiac sympathetic denervation for catecholaminergic polymorphic ventricular tachycardia. N Engl J Med.

[19] Schwartz PJ, Snebold NG, Brown AM (1976). Effects of unilateral cardiac sympathetic denervation on the ventricular fibrillation threshold. Am J Cardiol.

[20] Schwartz PJ, Stone HL (1982). Left stellectomy and denervation supersensitivity in conscious dogs. Am J Cardiol.

[21] Schwartz PJ (1984). The rationale and the role of left stellectomy for the prevention of malignant arrhythmias. Ann NY Acad Sci.

[22] Schwartz PJ, Priori SG, Zipes DP, Jalife J (2000). Sympathetic nervous system and cardiac arrhythmias. Cardiac electrophysiology: from cell to bedside.

[23] Schwartz PJ (2001). Another role for the sympathetic nervous system in the long QT syndrome?. J Cardiovasc Electrophysiol.

[24] Schwartz PJ, Stone HL (1979). Effects of unilateral stellectomy upon cardiac performance during exercise in dogs. Circ Res.

[25] Schwartz PJ (2010). Efficacy of left cardiac sympathetic denervation has an unforeseen side effect: medicolegal complications. Heart Rhythm.

[26] Goldenberg I, Bradley J, Moss AJ, McNitt S, Polonsky S, Robinson JL, Andrews M, Zareba W, International LQTS Registry Investigators (2010). Beta-blocker efficacy in high-risk patients with the congenital long-QT syndrome types 1 and 2: implications for patient management. J Cardiovasc Electrophysiol.

[27] Goldenberg I, Moss AJ, Peterson DR, McNitt S, Zareba W, Andrews ML, Robinson JL, Locati EH, Ackerman MJ, Benhorin J, Kaufman ES, Napolitano C, Priori SG, Qi M, Schwartz PJ, Towbin JA, Vincent GM, Zhang L (2008). Risk factors for aborted cardiac arrest and sudden cardiac death in children with the congenital long-QT syndrome. Circulation.

[28] Schwartz PJ, Spazzolini C, Crotti L (2009). All LQT3 patients need an ICD: true or false?. Heart Rhythm.

[29] Chatrath R, Bell CM, Ackerman M (2004). β-Blocker failures in symptomatic probands with genotyped long-QT syndrome. Pediatric Cardiol.

[30] Priori SG, Napolitano C, Schwartz PJ, Massimiliano G, Bloise R, Ronchetti E (2004). Association of long QT syndrome loci and cardiac events among patients treated with β-blockers. JAMA.

[31] Atkins HJB (1949). Peraxillary approach to the stellate and upper thoracic sympathetic ganglia. Lancet.

[32] Odero A, Bozzani A, De Ferrari GM, Schwartz PJ (2010). Left cardiac sympathetic denervation for the prevention of life-threatening arrhythmias: the surgical supraclavicular approach to cervicothoracic sympathectomy. Heart Rhythm.

[33] Gossot D, Kabiri H, Caliandro R, Debrosse D, Girard D, Grunenwald D (2001). Early complications of thoracic endoscopic sympathectomy: a prospective study of 940 procedures. Ann Thorac Surg.

[34] Ukena C, Bauer A, Mahfoud F, Schreieck J, Neuberger HR, Eick C, Sobotka PA, Gawaz M, Böhm M (2012). Renal sympathetic denervation for the treatment of electrical storm: first-in-man experience. Clin Res Cardiol.

[35] Rosso R, Kalman JM, Rogowsky O, Diamant S, Birger A, Biner S, Belhassen B, Viskin S (2010). Long-term effectiveness of beta blocker and calcium channel blocker combination therapy in patients with CPVT. Heart Rhythm.

[36] Sy RW, Gollob MH, Klein GJ, Yee R, Skanes AC, Gula LJ, Leong-Sit P, Gow RM, Green MS, Birnie DH, Krahn AD (2011). Arrhythmia characterization and long-term outcomes in catecholaminergic polymorphic ventricular tachycardia. Heart Rhythm.

[37] van der Werff C, Kannankeril PJ, Sacher F, Krahn AD, Viskin S, Leenhardt A, Shimizi W, Sumitomo N, Fish FA, Bhuiyan ZA, Willems AR, van der Veen MJ, Watanabe H, Laborderie J, Haissaguerre M, Knollmann BC, Wilde AA (2011). Flecainide therapy reduces exercised-induced ventricular arrhythmias in patients with catecholaminergic polymorphic ventricular tachycardia. J Am Coll Cardiol.

[38] Watanabe H, Chopra N, Laver D, Hwang HS, Davies SS, Roach DE, Duff HJ, Roden DM, Wilde AA, Knollmann BC (2009). Flecainide prevents catecholaminergic polymorphic ventricular tachycardia in mice and humans. Nat Med.

[39] Hilliard FA, Steele DS, Laver D, Yang Z, Le Marchand SJ, Chopra N, Piston DW, Huke S, Knollmann BC (2010). Flecainide inhibits arrhythmogenic Ca^2+^ waves by open states block of ryanodine receptor Ca^2+^ release channels and reduction of Ca^2+^ spark mass. J Mol Cell Cardiol.

[40] Schwartz PJ (2009). Cutting nerves and saving lives. Heart Rhythm.

[41] Svendsen JH, Geelen P, EHRA Scientific Initiative Commitee (2010). Screening for, and management of, possible arrhythmogenic syndromes (channelopathies/ion channel diseases). Europace.

[42] Veltmann C, Schimpf R, Borggrefe M, Wolpert C (2009). Risk stratification in electrical cardiomyopathies. Herz.

[43] Moray A, Krik EP, Grant P, Camphausen C (2011). Prophylactic left thoracic sympathectomy to prevent electrical storms in patients with CPVT needing ICD placement. Heart Lung Circ.

[44] Gopinathannair R, Olshansky B, Iannettoni M, Mazur A (2010). Delayed maximal response to left cardiac sympathectomy for catecholaminergic polymorphic ventricular tachycardia. Europace.

[45] Scott PA, Sandilands AJ, Morris GE, Morgan JM (2008). Successful treatment of catecholaminergic polymorphic ventricular tachycardia with bilateral thoracoscopic sympathectomy. Heart Rhythm.

[46] Makanjee B, Gollob MH, Klein GJ, Krahn AD (2009). Ten-year follow-up of cardiac sympathectomy in a young woman with catecholaminergic polymorphic ventricular tachycardia and an implantable cardioverter defibrillator. J Cardiovasc Electrophysiol.

